# Improving Measurement of Forest Structural Parameters by Co-Registering of High Resolution Aerial Imagery and Low Density LiDAR Data

**DOI:** 10.3390/s90301541

**Published:** 2009-03-04

**Authors:** Huabing Huang, Peng Gong, Xiao Cheng, Nick Clinton, Zengyuan Li

**Affiliations:** 1 State Key Laboratory of Remote Sensing Science, Jointly Sponsored by the Institute of Remote Sensing Applications of Chinese Academy of Sciences and Beijing Normal University, Beijing, 100101, P.R. China; E-Mails: huanghuabing@irsa.ac.cn; xcheng@irsa.ac.cn; nclinton@nature.Berkeley.edu; 2 Division of Ecosystem Science, University of California, Berkeley, CA 94720-3114, USA;; 3 Institute of Forest Resources Information Technology, Chinese Academic of Forestry, Beijing, 100091, P.R. China; E-Mail: zengyuan.li@forestry.ac.cn

**Keywords:** LiDAR, Aerial image, Forest structural parameters extraction

## Abstract

Forest structural parameters, such as tree height and crown width, are indispensable for evaluating forest biomass or forest volume. LiDAR is a revolutionary technology for measurement of forest structural parameters, however, the accuracy of crown width extraction is not satisfactory when using a low density LiDAR, especially in high canopy cover forest. We used high resolution aerial imagery with a low density LiDAR system to overcome this shortcoming. A morphological filtering was used to generate a DEM (Digital Elevation Model) and a CHM (Canopy Height Model) from LiDAR data. The LiDAR camera image is matched to the aerial image with an automated keypoints search algorithm. As a result, a high registration accuracy of 0.5 pixels was obtained. A local maximum filter, watershed segmentation, and object-oriented image segmentation are used to obtain tree height and crown width. Results indicate that the camera data collected by the integrated LiDAR system plays an important role in registration with aerial imagery. The synthesis with aerial imagery increases the accuracy of forest structural parameter extraction when compared to only using the low density LiDAR data.

## 1. Introduction

Forest structural parameters, such as tree height, crown width and canopy cover are critical to study the biomass, biogeochemical cycles, ecological function, exchange between mass and energy, water budgets and radiation transfer in a forest system [1–3]. Accurate forest inventory is crucial to forest resource management and wildlife habitat assessment [4,5].

It is desirable to use 3D measurement techniques to extract tree height and crown size information. Before airborne LiDAR became available, aerial photogrammetry and InSAR had been used to extract forest structural information at various scales ranging from individual trees to landscapes [6,7]. However, these 3D technologies require image matching from multi-angular images and it is often difficult to obtain reliable results. Preprocessing techniques that can help locate individual trees, particularly tree tops, are helpful to improve image matching accuracies. Wulder *et al*. adopted local maximum filtering to locate trees on high spatial resolution imagery [8]. Wang *et al*. used a marker-controlled watershed segmentation technique to extract crown size and detect treetops based on high spatial resolution aerial imagery [9]. However, these methods are based on optical imagery and assumed that treetops and crowns have higher reflected radiation because they expose more sunlit surface. Sometimes, under cloudy imaging conditions or with dense canopy closure, treetops are difficult to identify, even visually.

LiDAR (Light Detection and Ranging) is an active ranging technique that can directly measure 3D forest canopy coordinates at laser illuminated locations. Canopy coordinates can be used to characterize forest structural information [10]. A number of approaches have been proposed, with varying degrees of success, to characterize individual trees using LiDAR data. Bortolot adopted an object-oriented method using tree clusters as objects to assess canopy cover and density [11]. Popescu *et al*. used a local maximum filtering method with variable window size (based on a canopy height model) to extract tree height and used a regression model to retrieve crown diameter [12]. Koch *et al*. used a pouring algorithm to delineate crown shape based on treetops detected by local maximum filtering algorithm [13]. Chen *et al*. adopted watershed segmentation to isolate individual trees and proposed an improved watershed segmentation algorithm with a distance-transformed image to reduce inadequate segmentation [14]. All of these methods rely on computer vision techniques developed for optical imagery in order to process canopy height models. The density of the LiDAR point cloud affects the accuracy of crown shape delineation. Optical images provide information about geometry and color that is useful for delineation of tree crown shape and size. There is great potential for synergy between high-resolution optical imagery and LiDAR data for forest structural parameter extraction. However, only a small amount of research has been published on this topic.

Hill and Thomson used HyMap data to classify vegetation type and LiDAR data to retrieve canopy height [15]. Hudak *et al*. integrated Landsat Enhanced Thematic Mapper Plus (ETM+) and LiDAR to assess forest canopy height based on spatial and aspatial models [16]. Popescu and Wyne used high resolution optical imagery to differentiate deciduous trees and pines, and combined LiDAR data to estimate height of different tree species [17]. These studies provide evidence that integrating LiDAR data and optical imagery could improve extraction accuracy of forest structural parameters. The main problem in integrating high resolution optical imagery with LiDAR data is co-registration. Absolute geometric coordinate information has been used to match imagery. This method requires high accuracy navigation and tracking hardware, such as global positioning systems (GPS) and inertial measurement units (IMU) which describe the three dimensional orientation of the scanner according to the instrument pitch, roll and yaw.

The objective of this paper was to evaluate the quality, accuracy, and feasibility of an automatic tree extraction method based on low density LiDAR point data and high resolution imagery. Specific questions include:

How can LiDAR data be better registered with high resolution aerial imagery?
How much improvement in structural parameter extraction is possible when LiDAR data are integrated with high resolution aerial imagery?

Section 2 outlines the study area, LiDAR data specification, high resolution aerial image characteristics and field data. In Section 3, we introduce methods used for automatic DEM generation, registration and tree structural parameter extraction. The results are presented and discussed in Sections 4 and 5, respectively. Finally, some conclusions are drawn in Section 6.

## 2. Materials

### 2.1. Study area

**Figure 1.**
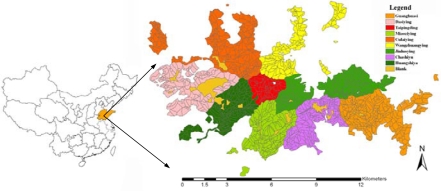
Location of Culai Mountain in Shandong Province, P.R. China.

The study area was selected in the Culai Mountain National Forest in the Shandong Province of China, with geographic coordinates 117°16′∼117°20′E, 36°02′∼36°17′N. This site is approximately 7,164 hectares and covers nine different forest types (Figure 1). The main tree species include hardwood (*Quercus liaotungensis*) and conifer trees (*Pinus armandi Franch*).

### 2.2. LiDAR and aerial imagery

The LiDAR data and the aerial imagery were collected May, 2005, using a Riegl LMS-Q280i airborne laser scanner and DCS22 (Digital Camera System 22 megapixels), respectively. The LiDAR was operated at a nominal altitude of 800 m above ground level and recorded the first returns as well as the return intensity in a single pass. A 22% overlap between adjacent strips ensured that no gaps appeared in the surveyed area. The maximum scan angles were ±30° off nadir and the average sampling space is about 1.6 m (0.43/m^2^ for whole area, 0.57/m^2^ for broadleaf tress and 0.65/m^2^ for conifer trees). The nominal accuracy of horizontal (x, y) and vertical (z) is about 0.5 and 0.2 m, respectively. The LiDAR contains a “true color channel” which provides, for each return, 8-bit red, green and blue (RGB) intensities of the target in addition to the x,y,z position information. Detailed specifications of the Riegl LMS-Q280i sensor can be found at the following URL: http://www.3dlasermapping.com/en/airborne/hardware/Q280i.htm. The DCS22 is a charge coupled device (CCD) camera with 22 mega pixels and each pixel is 9 μm in size. The DCS22 imagery has a 50 cm spatial resolution with a 40% overlap along the flying direction and a 30% overlap across flight lines.

### 2.3. Field data

We selected two different field sampling sites including hardwood and conifer trees and measured tree heights and crown widths using clinometers and tapes. Crown widths were estimated by averaging two direction measurements taken at North-South and West-East. The general statistical information of representative species is listed in table 1, which includes numbers, mean of tree height and crown width (Mean_TH and MD_CW) and standard deviation of tree height and crown width (SD_TH and SD_CW). The data in Table 1 were generated from field measurements of the trees. Tree positions were located with a GPS (LEICA GPS1200). Two GPS were used for location measurement, one for a base station and another for measuring. There are more than 20 GPS records for each tree position (www.geoservis.si/dnload/doc/System1200/GPS1200_ApplField_en.pdf).

## 3. Methods

### 3.1. LiDAR data processing

For the purpose of retrieving tree heights, a morphological filtering method was used to separate the ground points from the tree points and to generate the Digital Elevation Model (DEM). The CHM (Canopy Height Model) was created by subtracting the DEM from the Digital Surface Model (DSM). The morphological filtering algorithm was developed by Chen *et al*. [18]. The detailed procedure of DEM generation is as follows:

First, a grid was created to record the last return (the lowest z value) of all pulses falling in the cell. The size of grid was decided by the average scanning density:

(1) $$R=\sqrt{\frac{x\times y}{N}}$$

where *R* is the cell size in meters, *N* is the total number of returns in the point cloud and *x* and *y* are the dimensions in map coordinates.

If some cells had no return pulse within them, they were filled with the nearest cell value. This grid is denoted g_min_. Then, a morphological open operation was used to filter vegetation and artificial objects such as buildings; this grid is named g_open_. Finally, an initial set of terrain pulses were identified by calculating the difference between the grid after morphological filtering (g_open_) and the original grid (g_min_). The difference grid is denoted g_diff_. The cells of g_diff_ where |g_diff_| < Average(g_diff_) were classified as terrain pulses and these points were used to create a DEM with a Kriging interpolation.

### 3.2. CHM and aerial image registration

**Figure 2.**
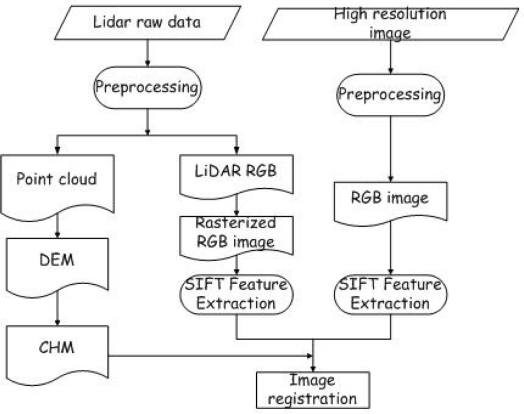
Flowchart of the image registration method.

The basic idea for registration of aerial images and the CHM is to use the RGB intensity information contained in the LiDAR records as a connection between the LiDAR data and the aerial image. In this LiDAR system, there is a true color channel that includes RGB intensity of the target with every return. So the LiDAR data not only include range information, but also reflected RGB information. The SIFT (Scale Invariant Feature transformation) algorithm was employed for matching LiDAR RGB data and aerial images. The SIFT was introduced by Lowe. It is an approach for detecting and extracting distinct features from images [19]. The features are invariant to image scale, rotation and robust with respect to changes in illumination, noise and, to some extent, changes in the 3D camera viewpoint. By using the feature points found by SIFT, a feature matching registration can be carried out. The flowchart of image registration is shown in Figure 2.

### 3.3. Tree height and Crown width retrieval

Treetop detection is a prerequisite for tree height and crown width retrieval. The basic assumptions of tree top detection using the CHM are as follows:

1. The tree top is always convex and surrounded by an area that is concave.
2. The canopy has a roughly circular outline when viewed from above.
3. The tree top has a higher value than the surrounding area in the CHM.
4. Trees are not clustered in such a way as to be confused for one crown.
5. There are an insignificant number of suppressed trees that are obstructed by a dominant overstory.

A local maximum filter algorithm was introduced to detect tree tops. The main problem encountered when using local maxima to detect tree tops is that non-treetop local maxima are incorrectly classified as treetops. A correlation relationship between tree height and crown size was used to reduce the commission errors, as described in Popescu and Wynne [17], and Chen *et al*. [14].

Watershed segmentation was used to extract crown size. The process of watershed segmentation can be illustrated in terms of flooding simulations [20]. Individual crown shape is retrieved after segmentation. There are two ways to calculate the crown size, one using a circle to fit crown shape and using the diameter as crown width, the other using average crown diameter along two perpendicular directions. In this paper, the circle fitting method was adopted. This method was more suitable for the low resolution CHM resulting from sparse LiDAR point clouds. An object-oriented segmentation method was used to process the high resolution aerial imagery to get the more elaborate crown information. This segmentation method uses region merging based on object heterogeneity of shape and spectral values [21]. The segmentation was performed using the BerkeleyImageSeg software package (http://www.imageseg.com/).

## 4. Results

**Figure 3.**
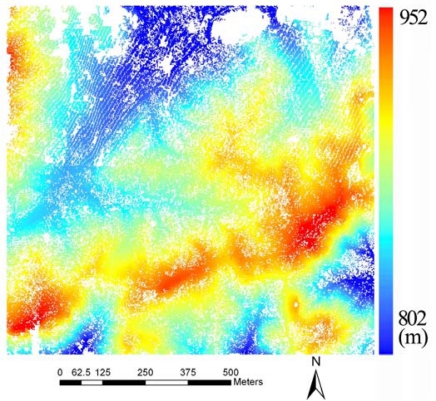
Initial DEM after morphological filtering.

Figure 3 shows the initial DEM after morphological filtering. The LiDAR points above ground have been removed leaving some gaps on the terrain.

**Figure 4.**
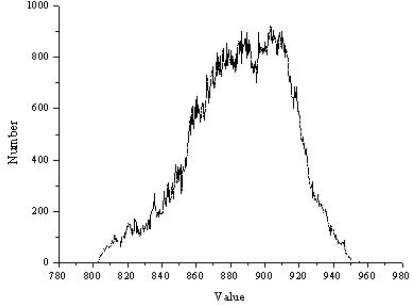
Distribution of the original DEM.

**Figure 5.**
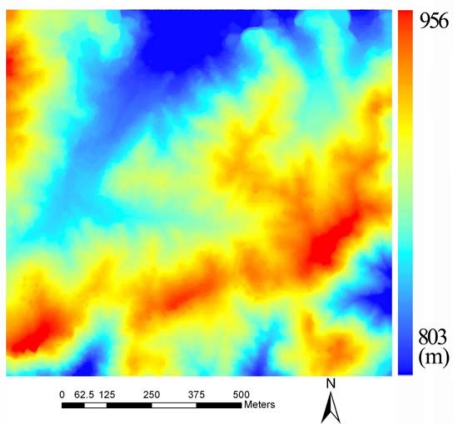
Final DEM after Kringing interpolation.

Figure 4 shows the frequency histogram of the initial DEM. As illustrated in Figure 4, the distribution of the original DEM is approximately normal, so Kriging interpolation was used to generate the final result as illustrated in Figure 5.

**Figure 6.**
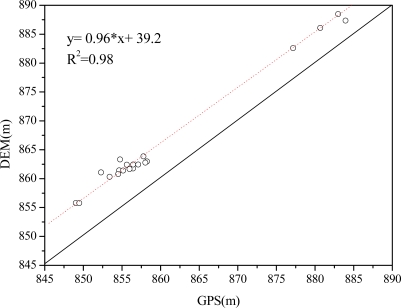
Correlation between measured GPS and DEM.

In order to assess the quality of the DEM, several points were measured in the field with high precision GPS. A Pearson’s correlation analysis (R), RMSE and MD (Equation 2) were performed to calculate the degree of correlation between the GPS measurements and the DEM (Figure 6):

(2) $$\mathrm{MD}=\mathrm{mean}(Y_{j}-Y_{i})$$

where *Y_j_* is the estimated measurement and *Y_i_* is the field measurement.

The R squared, RMSE and MD are 0.98, 4.41 and 3.95 m, respectively, and the regression curve is almost parallel to the one to one line. This indicates that the DEM is close to elevation measured by GPS but has a systematic error in it.

**Figure 7.**
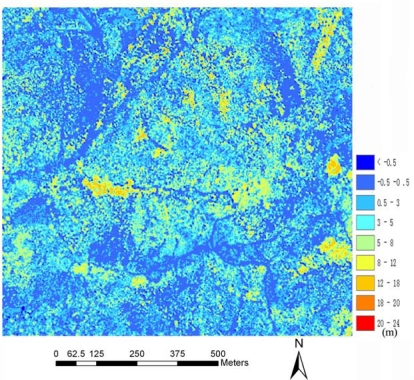
The CHM generated from LiDAR data.

The canopy height model is shown in Figure 7. As shown as Figure 7, most points have values from −0.5 to 0.5 m. Greater values were colored by yellow and red, which indicate trees in the CHM.

**Figure 8.**
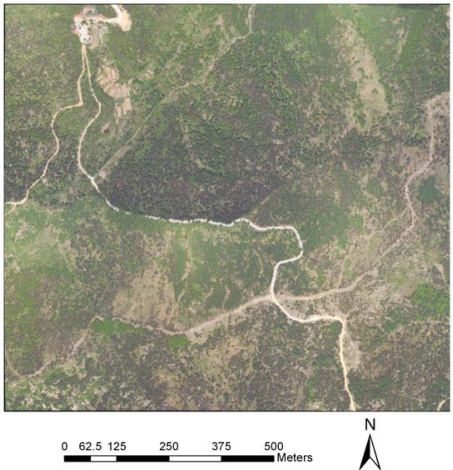
Aerial image covering the study area.

**Figure 9.**
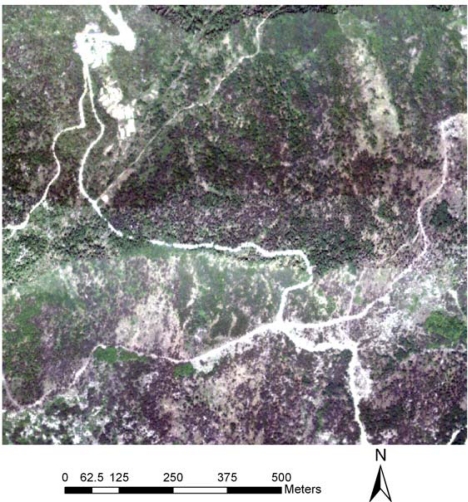
True color image rasterized from the RGB intensity.

Figures 8 and 9 show high resolution airborne imagery and true color imagery rasterized from the RGB intensity of LiDAR returns, respectively. 141 pairs of control points were selected using the SIFT algorithm and a cubic polynomial was used to match the rasterized RGB to the high resolution airborne imagery.

**Figure 10.**
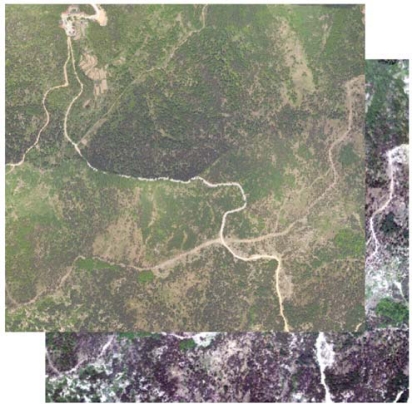
Relative position before image registration.

**Figure 11.**
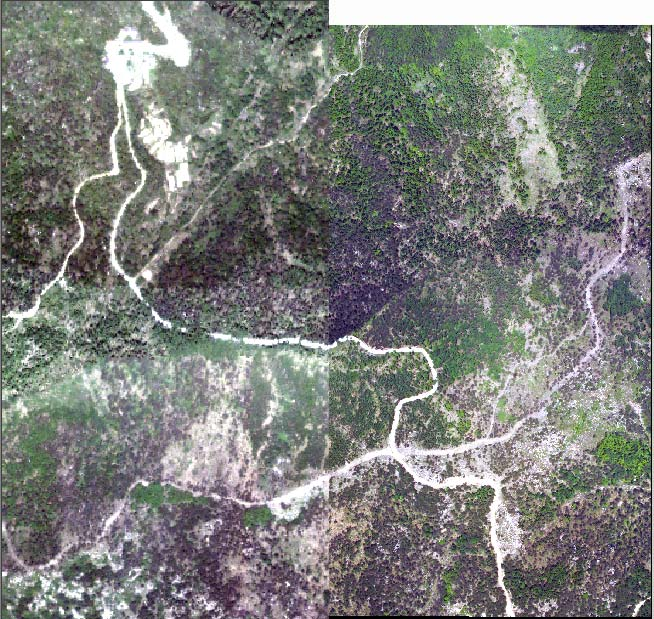
Local slice after image registration.

Figures 10 and 11 show their relative positions before registration and after registration, respectively. As shown in Figure 11, we can see that roads connect after registration. In order to assess the match accuracy, 20 points was selected randomly, and the RMSE computed from the points is 0.47 pixels, indicating a good match.

**Figure 12.**
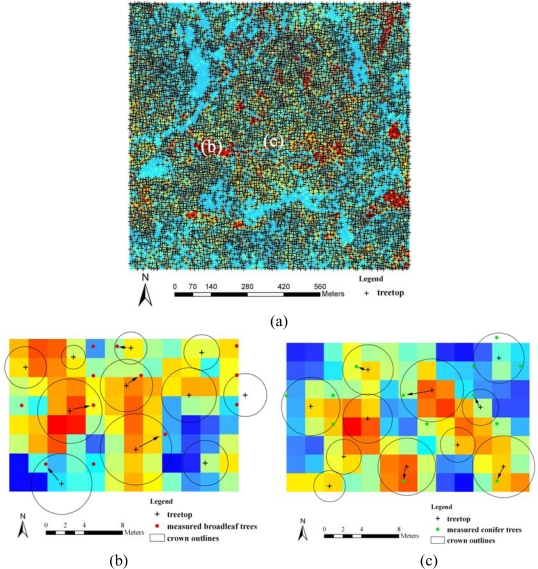
(a): Tree tops obtained from applying local maximum filtering to CHM, (b) and (c): Larger scale view of the tree tops (shown with cross) and ground measured trees (dot) of broadleaf and conifer trees, respectively, where the matched trees are linked with arrows.

Figure 12 shows location of treetops using the local maximum filter and trees are paired by visual analysis. Tree positions measured in the field were compared to the results obtained from the CHM using local maximum filtering and from the aerial image using object-oriented segmentation. Each GPS measured tree was paired with a corresponding treetop when a GPS measured tree location was within the coverage of a crown diameter estimated by watershed segmentation from the CHM or object-oriented segmentation from the aerial image. Errors of omission were recorded when a field-measured tree could not be successfully paired with a tree identified from the CHM or the aerial image. Errors of commission were not recorded because not all of the trees were measured in the field [22].

Table 2 presents omission errors for broadleaf and conifer trees identified from the CHM and aerial image. As listed in Table 2, omission errors from the CHM are greater than those from the aerial image for both broadleaf and conifer trees. Omission errors from the CHM have a greater difference between the two species (57.14% for broadleaf trees and 75% for conifer trees) than those from the aerial image (45.71% for broad leaf trees and 51.78% for conifer trees). As a whole, broadleaf trees could be identified better than conifer trees with both methods.

**Figure 13.**
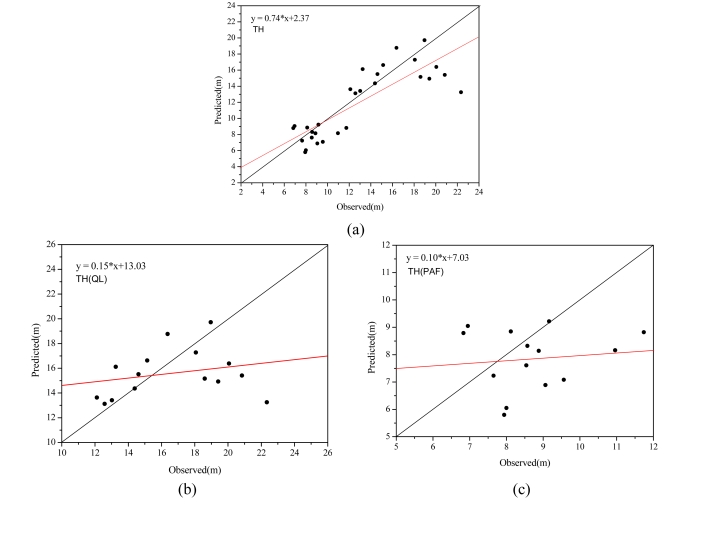
Scatter plot between observed tree heights and those retrieved from CHM, (a) for overall trees, (b) for broadleaf trees and (c) for conifer trees.

Pearson’s correlation (R), RMSE and MD analysis between measured tree height and predicted tree height is illustrated in Table 3 and Figure 13. In order to analyze tree height extraction of different species, tree height statistics for both broadleaf and conifer trees are illustrated. As shown in Table 3 and Figure 13, tree heights for conifers and broadleaves are in distinct categories, which contribute to a high correlation when the trees are combined. The R coefficient, RMSE and MD are 0.83, 2.78 and −0.97 m, respectively, though most predicted values are lower than measured values (−0.97 m). As for individual species, tree heights of conifer trees have a smaller RMSE and MD (1.81 m and −0.85 m) than those of broadleaf trees (3.45 m and −1.07 m). However, the correlation between measured and predicted tree heights of broadleaf is greater than that of conifer trees (0.26 for broadleaf trees and 0.12 for conifer trees).

**Figure 14.**
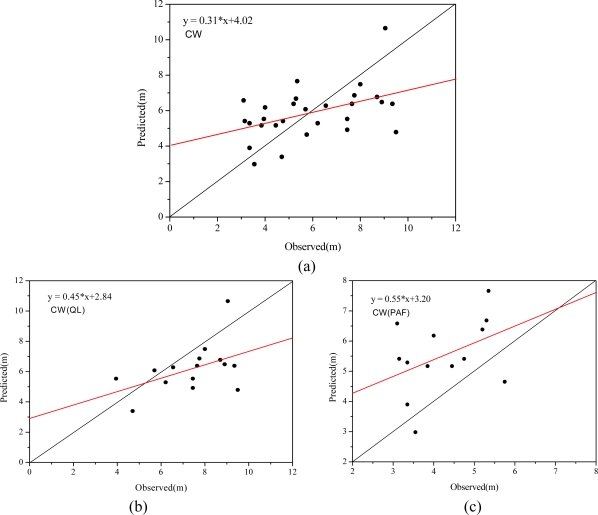
Comparison of observed tree crown widths with those retrieved from CHM using watershed segmentation, (a) for overall trees, (b) for broadleaf trees and (c) for conifer trees.

Goodness-of-fit statistics and scatter plots between measured and predicted crown width from the CHM for broadleaf, conifer and combined trees are shown in Table 4 and Figure 14, respectively.

As listed in Table 4, the R coefficient, RMSE and MD are 0.46, 1.88 m and −0.01 m, respectively, for combined species. Goodness-of-fit statistics of broadleaf and conifer are compared in Table 4. The two species have similar correlation, 0.47 for broadleaf trees and 0.43 for conifer trees. Crown widths of conifer trees have a smaller RMSE (1.71 m) than those of broadleaf trees (2.02 m). As the results in Table 4 show, most extracted crown widths of broadleaf trees were less than observed crown widths (MD is −1.21 m) and those of conifer trees were greater than measured crown widths (MD is 1.26 m). Comparison of Figure 14 (b) and (c) also shows a similar result.

**Figure 15.**
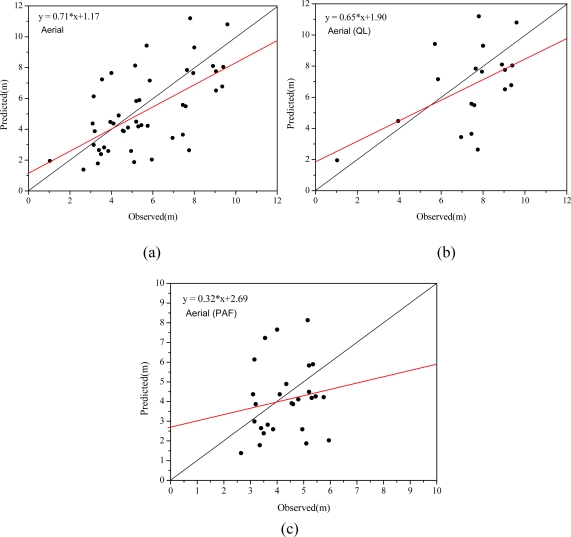
Scatter plot of measured crown widths and those extracted from high resolution aerial image, (a) for overall trees, (b) for broadleaf trees and (c) for conifer trees.

Table 5 and Figure 15 show statistics and scatter plots of measured crown width and crown width determined from object-oriented segmentation on the high resolution imagery for overall trees, broadleaf and conifer trees. More trees were identified and paired with trees measured in the field, resulting in 19 broadleaf trees and 27 conifer trees matched. The comparison between Table 4 and Table 5 shows that the correlation coefficient increases from 0.46 to 0.61 and more points in Figure 15 are closer to the one to one line. This comparison indicates that higher accuracy of crown width extraction from segmentation of the aerial imagery than from the CHM. As for individual species, correlation between measured and predicted crown widths increases from 0.47 to 0.51 for broadleaf trees, while it decreases from 0.43 to 0.17 for conifer trees. Mean difference is reduced from −1.21 to −0.67 for broadleaf trees and from 1.26 to −0.21 for conifer trees. The two different crown width retrieval methods have divergent results for conifer trees. Predicted crown width from CHM overestimated actual crown width, while those from aerial image underestimated actual crown width.

## 5. Discussion

Airborne LiDAR data is often used to provide detailed information on tree canopy structure. Lower accuracy was obtained when using low density LiDAR data, as it is difficult to capture crown shape with low density LiDAR data [22]. As more and more airborne LiDAR systems integrate with CCD cameras, it is highly relevant to assess methods that can combine LiDAR and high resolution imagery for forest structural information extraction. This process is constrained by difficulties in co-registration of airborne LiDAR range data and aerial images. Using the additional RGB intensity included in the LiDAR data, the method proposed here serves as a bridge to match LiDAR range data and high resolution aerial imagery. In this paper, a SIFT algorithm was introduced to automatically find tie points and a cubic polynomial was used to perform registration based on the tie points. The method presented in this paper increased the crown width extraction accuracy when compared with crown width extraction result only based on the CHM, especially for broadleaf trees. More trees were identified and paired with measured trees, and the correlation increases from 0.46 to 0.61. More and more LiDAR systems include intensity information reflected by objects. This intensity could play an important role in co-registration of LiDAR range data and other images. The methods proposed here could also be effective for matching multi-temporal LiDAR data for the purposes of forest growth detection.

The LiDAR point cloud filtering is of primary importance for CHM generation. The morphological filtering algorithm used in this paper achieved mixed results when compared to field estimated values. While the extracted heights seemed to underestimate the field observed values (especially for broadleaf trees), it is just as valid to assume that the field observations over-estimated tree heights. As field estimation of tree heights can be difficult and notoriously inaccurate, we feel it is prudent to exercise caution in using field observed values as “ground truth.” Our results indicate disagreement between LiDAR extracted heights and field estimated heights, but it is difficult to determine which data set is more accurate. It may be possible to increase the accuracy of LiDAR height extraction by incorporating additional information to the automatic filtering algorithm. The filter could be more adaptive by incorporating intensity and contextual information from aerial images and LiDAR point cloud data. Another way to increase accuracy of the LiDAR based tree height extraction is to increase the sampling frequency of the LiDAR. A greater number of LiDAR returns per unit area would result in a denser LiDAR point cloud from which a more accurate CHM can be interpolated.

Although we have demonstrated that combining high resolution aerial imagery with LiDAR can make up for some of the limitations of a low density LiDAR point cloud, both crown widths from CHM and aerial image were poorly correlated with field measured crown widths. Low density of data is one potential source of error, while tree density, models of crown shape, and surface generation also affect accuracy. We estimated the average tree spacing in the study area at about 6 meters. In higher density forests, it is possible that this method would have higher error. The methods we describe (watershed segmentation for CHMs and object-oriented segmentation for aerial imagery) are more effective for dominant tree detection than co-dominant and suppressed trees. Additional research is needed to explore more effective crown width and tree height extraction methods, especially for clustered and/or suppressed trees. There is also a need to establish the relationship between LiDAR point cloud density and the accuracy of extracted forest structural parameters.

## 6. Conclusions

We describe a method for integrating sparse LiDAR data and high resolution aerial imagery to extract forest structural parameters. A morphological filtering algorithm was effective to pre-process the LiDAR point cloud and a Kriging interpolation was successful for generating a digital elevation model (DEM) and canopy height model (CHM). We also found local maximum filtering to be effective for detection of individual treetops. Inclusion of additional intensity information within a LiDAR system helps with bridging LiDAR range data and high resolution aerial imagery during geometric registration. The SIFT algorithm was harnessed for this purpose with good results. Watershed segmentation and object-oriented methods were successfully used to extract crown width based on CHM and high resolution aerial imagery. From our results, we conclude that accuracy of forest structural extraction can be improved combining high resolution imagery with LiDAR data.

## Figures and Tables

**Table 1.** Statistical information of trees measured in the field, by species.

**Species**	**Numbers**	**Mean_TH(m)**	**SD_TH(m)**	**Mean_CW(m)**	**SD_CW(m)**
*Quercus liaotungensis*	35	16.81	3.54	7.37	1.91
*Pinus armandi Franch*	56	8.96	1.70	4.30	1.20

**Table 2.** Omission errors of broadleaf and conifer trees identified from CHM and aerial images.

**Species**	**CHM**	**Aerial image**
*Quercus liaotungensis* (QL)	57.14%	45.71%
*Pinus armandi Franch* (PAF)	75.00%	51.78%

**Table 3.** Goodness-of-fit statistics between observed tree heights and those predicted from CHM.

**Numbers**		**Correlation**		**RMSE(m)**		**MD(m)**	
QL	PAF	QL	PAF	QL	PAF	QL	PAF
29		0.83		2.78		−0.97	
15	14	0.26	0.12	3.45	1.81	−1.07	−0.85

**Table 4.** Goodness-of-fit statistics between observed crown widths and those predicted from CHM.

**Numbers**		**Correlation**		**RMSE(m)**		**MD(m)**	
QL	PAF	QL	PAF	QL	PAF	QL	PAF
29		0.46		1.88		−0.01	
15	14	0.47	0.43	2.02	1.71	−1.21	1.26

**Table 5.** Goodness-of-fit statistics between observed crown widths and those predicted from Aerial image.

**Numbers**		**Correlation**		**RMSE(m)**		**MD(m)**	
QL	PAF	QL	PAF	QL	PAF	QL	PAF
46		0.61		2.10		−0.40	
19	27	0.51	0.17	2.40	1.86	−0.67	−0.21
